# Effects of physical activity on academic burnout among rural left-behind children in China: the chain-mediated roles of loneliness and general self-efficacy

**DOI:** 10.3389/fpsyg.2025.1653243

**Published:** 2025-10-23

**Authors:** Jiaxi Chen, Xiao Liu, Jiaqi Xie, Qian Yang, Ziping Fan, Dianhui Peng, Yujuan Wang, Chunxia Lu

**Affiliations:** ^1^Hunan Normal University, Changsha, China; ^2^Hunan University of Finance and Economics, Changsha, China

**Keywords:** physical activity, academic burnout, loneliness, general self-efficacy, rural left-behind children

## Abstract

**Purpose:**

To explore the impact of physical activity on academic burnout among rural left-behind children.

**Methods:**

In total, 195 rural left-behind children of Furen Primary School in Yongzhou City by combining stratified and cluster sampling methods. Participants completed the Physical Activity Scale, the Academic Burnout Scale, the Loneliness Scale, and the General Self-efficacy Scale.

**Results:**

(1) Empirical evidence indicates alarming rates of academic burnout among rural left-behind children in China. (2) Physical activity was significantly associated with reduced academic burnout. Our serial mediation model showed that physical activity had a significant direct effect on academic burnout (*β* = −0.231, 95% CI = [−0.308, −0.154]) in addition to significant chain-mediated effects on academic burnout via loneliness and general self-efficacy (*β* = −0.051, 95% CI = [−0.093, −0.018]); the more exercise participation, the lower the academic burnout among rural left-behind children. (3) These findings suggest that physical activity is an important interventional target when aiming to reduce academic burnout.

**Conclusion:**

Physical activity can affect rural left-behind children’ academic burnout both directly and through mediating effects. Loneliness and general self-efficacy partially mediate the impact of physical activity and academic burnout.

## 1 Introduction

As China continues its rapid urbanization and industrial development, a significant portion of rural workers have relocated to cities in search of employment opportunities. However, due to various socioeconomic constraints, many of these migrant laborers are unable to bring their children with them, resulting in a large population of minors remaining in their rural hometowns. These youth have become widely recognized in academic and policy circles as “left-behind children” (LBC), defined as children aged 0–16 who have their parents or one parent moving across the township street for more than half a year and stay in their original home and cannot live with their parents (National Bureau of Statistics of China, 2017). In China, approximately 40 million (38%) rural children are left behind by one or both migrant parents, accounting for approximately 15% of all children in the entire country (National Bureau of Statistics of China, 2020). Left-behind children are confronting a spectrum of escalating challenges in psychological and educational domains, with profound implications for the development of severe academic burnout. This emerging crisis has drawn increasing attention to the psycho-social and educational vulnerabilities of these children. Academic burnout manifests through three primary dimensions: (1) physical and mental health deterioration (e.g., chronic fatigue, sleep disturbances); (2) emotional dysregulation (e.g., persistent anxiety, depressive symptoms); and (3) maladaptive behaviors (e.g., increased aggression, school dropout rates). In extreme cases, these cumulative stressors have led to suicidal ideation and attempts, highlighting the urgent need for systemic interventions (Yu et al., 2023; Arbabisarjou et al., 2015). The academic burnout of left-behind children has become a hot topic of social concern.

Recent studies have begun to unravel the complex relationship between physical activity and academic burnout, suggesting that regular engagement in physical activities may mitigate the symptoms of academic pressure (Chen K. et al., 2022). The adverse effects of sustained academic pressure include academic burnout, a syndrome manifesting as exhaustion, cynicism, and impaired academic performance (Gao, 2023). However, the intrinsic mechanism of the effect of physical activity on academic burnout in rural left-behind children has still not been clearly studied. Loneliness, is defined as subjective distress from perceived deficiencies in social relationships. While typically transient, chronic or severe loneliness—which is pervasive among left-behind children due to lacking familial support and social integration—can negatively impact their health and well-being (Feng et al., 2024), has been identified as a significant correlate of academic burnout (Jiang et al., 2020). Concurrently, general self-efficacy, the belief in one’s capabilities to organize and execute actions required to manage prospective situations (Chen K. et al., 2022), emerges as a pivotal factor in coping with academic challenges and in fostering resilience against burnout.

Therefore, we hypothesize that physical activity may directly influence academic burnout but also indirectly through its impact on loneliness and general self-efficacy. Specifically, we propose that physical activity could reduce the feeling of loneliness, which in turn could enhance general self-efficacy, thereby collectively alleviating academic burnout (Feng et al., 2024). A comprehensive understanding of these relationships is essential for designing evidence-based interventions that harness physical activity as a protective factor to enhance the academic resilience of rural left-behind children. By elucidating the mediating mechanisms of loneliness and general self-efficacy, we can identify critical pathways through which physical activity mitigates academic burnout, thereby informing more effective support strategies for this vulnerable population. Failure to address these issues may result in: (1) exacerbated academic burnout, (2) progressive mental health deterioration, (3) impaired social adaptation, and (4) persistent developmental disadvantages. Such outcomes would not only perpetuate intergenerational educational disparities but also impose significant long-term socioeconomic burdens. By examining the mechanisms through which physical activity may influence academic burnout via loneliness and general self-efficacy, this study seeks to generate actionable insights. The findings are expected to offer evidence-based guidance for researchers, education policymakers, and mental health professionals in formulating targeted interventions to support left-behind children.

### 1.1 Mediating role of loneliness

Loneliness represents a critical psychological state that individuals may experience when they feel socially isolated or lack a sense of connection (Zhao et al., 2024). And that can significantly impact on children’s emotional and social well-being, especially for left-behind children who may experience a lack of parental care and companionship. Research has established a link between loneliness and the frequency of physical activity among primary schools (Rangul et al., 2024). Students who engage less frequently in physical activity tend to experience higher levels of loneliness, which in turn affects their ability to cope with challenges effectively (Zhao et al., 2024). Moreover, loneliness is a significant variable influencing academic burnout (Wang et al., 2023). Students who are less lonely and more connected with their peers are more likely to adopt a problem-solving approach rather than an emotionally oriented one (Zhao et al., 2024). Loneliness exerts a bidirectional influence on physical-activity trajectories and, by extension, on the emergence of academic burnout (Zhao et al., 2024). Lonely students—harboring negative self-schemas and social disconnection—withdraw from movement contexts, thereby depriving themselves of a potent buffer against burnout. Conversely, diminished loneliness predicts sustained physical-activity engagement, which reinforces adaptive coping and attenuates burnout risk. This recursive pattern is consistent with the evolutionary account of loneliness, wherein maladaptive cognitions and perceived relational deficits potentiate a self-perpetuating cycle of avoidance and exhaustion (Wu et al., 2024). Parental absence and attenuated social scaffolding render rural left-behind children acutely susceptible to loneliness. Elevated loneliness, in turn, activates negative self-schemas in which children construe themselves as incapable of satisfying social and academic demands, thereby precipitating maladaptive self-evaluations (Khurshid et al., 2025). As a result, loneliness may hinder children from building meaningful relationships and engaging in academic activities, thereby promoting the experience of academic burnout.

### 1.2 Mediating role of general self-efficacy

General self-efficacy, the belief in one’s ability to succeed in various tasks (Alhadabi and Karpinski, 2020), is crucial for how students handle academic challenges and stress, affecting their academic performance (Mohammadyari, 2012). It has been shown that the high general self-efficacy students are more likely to engage in physical activity, seeing it as a manageable task, which can lead to better mental health and stress management (Zhang et al., 2024). This engagement can reduce academic burnout, as these students can better cope with academic demands and stay motivated (ÖZhan, 2021). In contrast, students with low general self-efficacy may avoid physical activity, increasing their risk of burnout. Studies show that general self-efficacy can mediate the link between physical activity and mental health (Adams et al., 2020), influencing individuals benefit from physical activity. For left-behind youth, heightened general self-efficacy is theorized to galvanize physical activity engagement, which subsequently attenuates academic burnout. This segment interrogates a serial mediation model in which general self-efficacy functions as the second-stage mediator linking physical activity to diminished burnout, drawing on evidence that underscores the burnout-mitigating potency of this cognitive resource.

### 1.3 Relationship between loneliness and general self-efficacy

Loneliness, as a psychological state characterized by feelings of isolation and a lack of social connection, can significantly impact a child’s overall well-being and mental health (Kwan et al., 2020). General self-efficacy, on the other hand, refers to an individual’s belief in their capabilities to successfully perform tasks and influence outcomes across various situations (Wood et al., 2022). A robust inverse association links loneliness to general self-efficacy: as loneliness intensifies, perceived competence declines, eroding children’s capacity to manage everyday stressors. Among China’s rural left-behind youth, parental absence amplifies this dynamic, rendering them exceptionally vulnerable. A recent meta-analysis confirms that separation from parents is a primary driver of elevated loneliness and concomitant psychological distress in this population (Jia and Tian, 2010). The sequelae of parental absence extend beyond loneliness to encompass depression, anxiety, and suicidal ideation conditions are amplified when self-efficacy is low. General self-efficacy functions as a critical buffer, mediating the pathway from loneliness to diminished life satisfaction. Children who appraise themselves as efficacious report higher life satisfaction, self-esteem and positive affect, whereas those with attenuated self-efficacy are disproportionately exposed to loneliness, depressive symptoms, and negative effect. The loneliness and general self-efficacy nexus is thus bidirectional, mutually reinforcing. Interventions that simultaneously attenuate loneliness and cultivate self-efficacy are imperative for interrupting this cycle, mitigating the psycho-social costs of parental absence, and fostering adaptive psychological functioning among left-behind primary-school students.

### 1.4 Present study and hypotheses

**Figure 1:**
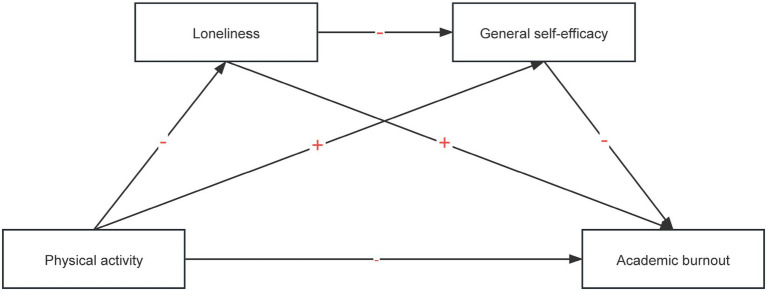
A Hypothetical model of the influence of physical activity and academic burnout.

While extant scholarship has documented bivariate links among physical activity, academic burnout, loneliness, and general self-efficacy, these constructs have typically been treated as discrete predictors. Consequently, the literature remains fragmented, and no integrative inquiry has yet articulated their systematic, joint influence on academic burnout (Chen K. et al., 2022). This study set out to map how loneliness and general self-efficacy sequentially translate variation in physical activity into differences in academic burnout among elementary students. By positioning these two constructs as serial mediators, we aimed to uncover the fine-grained psychological processes that drive school-related exhaustion and to furnish a theory-driven platform for school-based mental-health initiatives. We proposed the following hypotheses (hypothesized model is shown in Figure 1).

*H1: Physical activity directly affects the academic burnout of rural left-behind children.*

*H2: Loneliness mediates the relationship between physical activity and the academic burnout of rural left-behind children.*

*H3: General self-efficacy mediates the relationship between physical activity and the academic burnout of rural left-behind children.*

*H4: Loneliness and general self-efficacy play chain-mediating roles in the relationship between physical activity and academic burnout.*

## 2 Design

### 2.1 Participants and context

**Figure 2:**
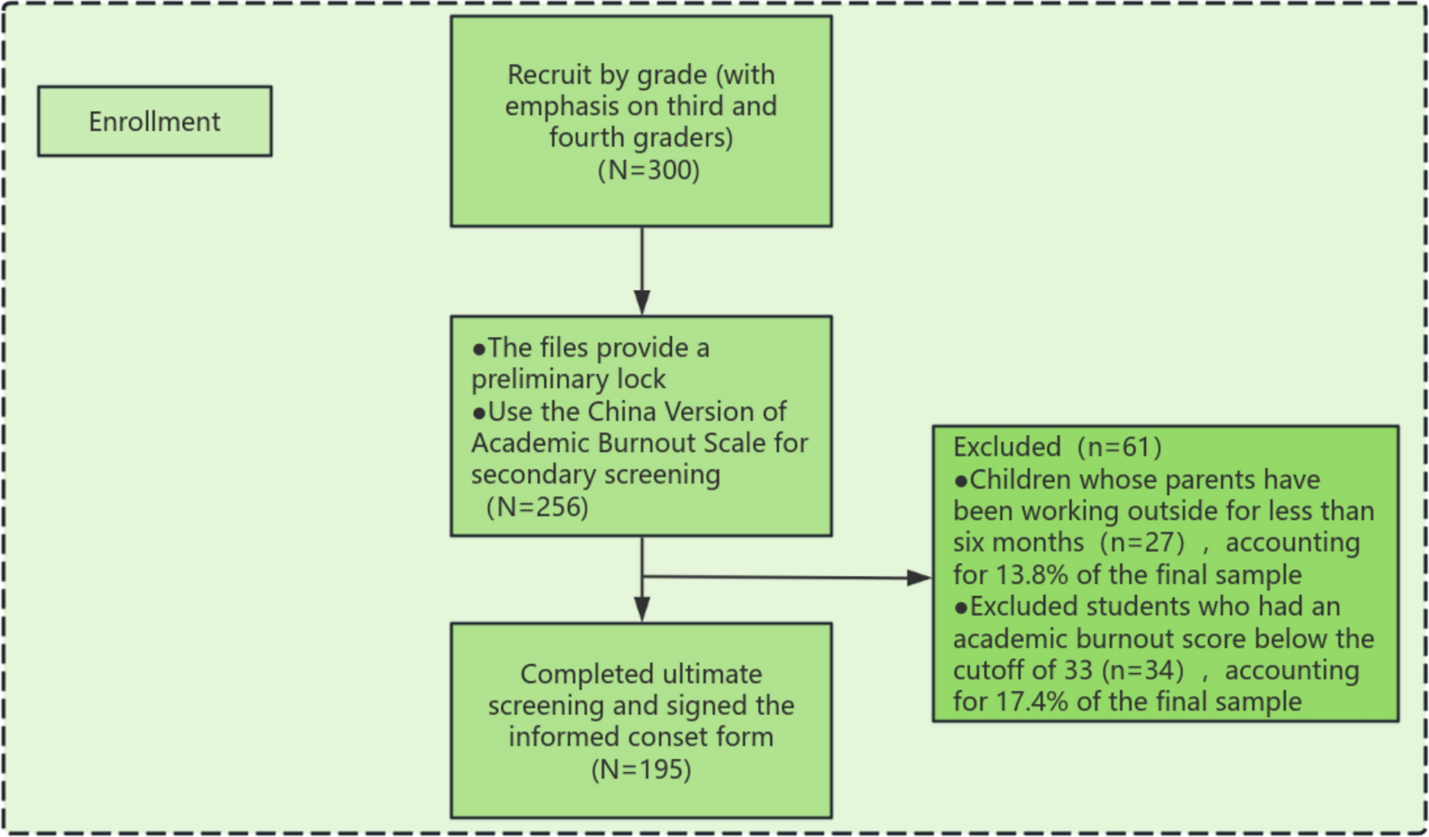
Recruitment process flow chart.

From March to June 2023, this study conducted a questionnaire survey on students of Furen Primary School in Yongzhou City by combining stratified and cluster sampling methods. The sample was first stratified by grade (focusing primarily on Grade 3 and 4) and academic burnout level. Cluster sampling was then conducted at the class level to identify students who met the criteria for left-behind children—defined as those with both parents working outside the home for at least 6 months. Potential participants were initially identified through school records, and further screened using the “The Chinese version of the Academic burnout Scale.” The study specifically sampled students exhibiting academic burnout, excluding those with scores below the minimum threshold of 33 on the academic burnout scale. As illustrated in Figure 2, these excluded students accounted for 17.4% of the final sample pool. The study sample comprises 195 individuals, with a gender distribution of 107 males (54.8%) and 88 females (45.1%). Age distribution is categorized into two groups: 7–8 years with 81 individuals (41.4%) and 9–10 years with 114 individuals (58.2%). In terms of grade, the sample is divided into two groups: Grade 3 with 84 individuals (42.9%) and Grade 4 with 111 individuals (56.7%). Regarding parental marital status, the majority of the sample comes from married households, accounting for 151 individuals (77.4%). The remaining proportions are as follows: parental dissociation for 34 individuals (17.4%), bereft of one’s spouse for 5 individuals (2.5%), and remarry for 5 individuals (2.5%). In terms of guardianship, the sample is primarily divided between those under the care of paternal grandparents with 91 individuals (46.6%) and those under the care of maternal grandparents with 101 individuals (51.7%). A small proportion of 3 individuals (1.5%) are under the care of other types of guardianship, as shown in Table 1.

**Table 1:** The demographics of the participants.

Variable	Category	Frequency (*n* = 195)	Ratio
Student gender	Male	107	54.8
	Female	88	45.1
Age(years)	7–8	81	41.4
	9–10	114	58.2
Grade	3	84	42.9
	4	111	56.7
Parents marriage	married	151	77.4
	dissociaton	34	17.4
	bereft of one’s spouse	5	2.5
	remarry	5	2.5
Type of guardianship	Paternal grandparents	91	46.6
	Maternal grandparents	101	51.7
	other	3	1.5

### 2.2 Procedures

This research adhered to rigorous ethical and methodological protocols. To minimize response bias, the survey instrument integrated items from different constructs in an interleaved sequence. Before administration, all participants received a clear verbal overview of the study’s aims, procedures, and scientific relevance. Particular emphasis was placed on voluntary involvement and the unconditional right to withdraw at any stage, considering the vulnerable background of the target population. To enhance response authenticity and protect confidentiality, full anonymity and stringent data security measures were implemented throughout the process. Informed written consent was secured prior to survey initiation. Participants completed the questionnaire individually in a supervised setting, with an average duration of 20 min. All responses were collected promptly upon completion. To ensure procedural uniformity and adherence to ethical guidelines, all research staff participated in a standardized training regimen before engaging with participants.

### 2.3 Methods

#### 2.3.1 Physical activity scale

To evaluate the physical activity levels of individuals, the well-established Physical Activity Rank Scale (PARS-3) developed by Liang (1994) was utilized. This scale has been widely used among Chinese adolescents (Bai et al., 2022), particularly in cross sectional studies. The scale operationalizes physical activity along three graded dimensions—intensity (“How intensely do you exercise?”), duration, and frequency—each subdivided into five ascending levels (intensity and frequency: 1–5; duration: 0–4). Total engagement is quantified by the product Intensity × Duration × Frequency, yielding a continuous metric bounded by 0 and 100, wherein higher values denote greater overall activity. In this study, the measure exhibited good reliability and validity, as indicated by Cronbach’s alpha was 0.740 and KMO value was 0.712.

#### 2.3.2 Academic burnout scale

The Chinese version of the Academic burnout scale, developed by Wu et al. (2010), was employed in this study. The 16-item inventory captures academic burnout across three theoretically derived facets: physical/mental exhaustion (e.g., “I feel utterly drained by the end of the school day”), academic alienation (e.g., “I”m so incompetent at schoolwork that I want to quit”), and diminished accomplishment (e.g., “I consistently underperform on exams”). Items are rated on a 5-point Likert continuum (“1″ = strongly disagree, “5″ = strongly agree), with higher aggregate scores denoting elevated burnout severity. Prior to administration, all items were reviewed by experts and rural teachers to ensure cultural and linguistic appropriateness for the target population, thereby minimizing contextual misunderstanding. The current study found that the measure had good reliability and validity, evidenced by Cronbach’s alpha was 0.931 and KMO value was 0.918.

#### 2.3.3 Loneliness scale

The Children’s Loneliness Scale (CLS) is used to measure the level of loneliness among LBC (Asher et al., 1984). The Children’s Loneliness Scale (CLS) comprises 24 statements. Sixteen core items gauge perceived loneliness and social dissatisfaction; eight filler items on hobbies and daily activities are interspersed to reduce response fatigue. Each item is rated on a five-point continuum anchored by “Not true at all” (1) and “Always true” (5). Scores are derived from the 16 core items, yielding a continuous metric from 16 to 80. Original cut-points classify respondents as non-lonely (≤ 27), moderately lonely (28–36), highly lonely (37–45), or severely lonely (≥ 46). To improve cultural applicability for rural left-behind children in China, the scales underwent expert review by educational psychologists and rural teachers. Items were linguistically and contextually adapted—such as specifying “peers” as “classmates” or “friends in the village”—to enhance relevance and comprehension. The CLS has proved to be the most suitable scale for assessing children’s loneliness (Cole et al., 2021), and in the present study it showed good reliability and validity (Cronbach’s ɑ = 0.890, KMO = 0.837).

#### 2.3.4 General self-efficacy scale

The General Self-Efficacy Scale (GSES) was used to assess the general self-efficacy of Rural Left-Behind Children (Zhang and Schwarzer, 1995). The ten-item scale is anchored on a 4-point Likert continuum with 1 being never and 4 being very often; a representative item reads, “If I try my best, I can always solve the problem.” Scores are summed across items, with higher aggregates denoting stronger general self-efficacy. Scale items were culturally and contextually adapted to enhance suitability for rural left-behind children without compromising core measurement validity. Adaptation strategies drew upon regionally relevant literature and were approved by specialists in educational psychology. Extensively validated across Chinese samples, the measure demonstrates robust psychometric properties (Chen X. et al., 2022; Cheng et al., 2020). In the present study, the measure demonstrated good reliability and validity, with Cronbach’s alpha was 0.944 and KMO value was 0.952.

### 2.4 Data analysis

First, we coded the questionnaire data after excluding questionnaires with obvious regular responses as well as those with obvious inconsistencies in the answers. The coded data were examined using descriptive statistics and underwent correlation analysis using SPSS 27.0. AMOS 26.0 software was used for validation factor analysis. Next, we established the chain mediation model. The mediation effect test was conducted using a bootstrap method with a sampling number of 5,000; when the 95% confidence interval did not include 0, it indicated a mediating effect. Significance levels are indicated as follows: ^*^p < 0.05, ^**^p < 0.01, ^***^p < 0.001.

## 3 Results

### 3.1 Common method bias test

Because the data in this study were collected through self-report measures from participants, the common method variance (CMV) may exist. Harman’s single-factor method analysis was conducted to assess the severity of data homology errors in this study (Hong-xing et al., 2012). The results showed that there were eight common factors with explained greater than 1, and the first common factor explaining the variance accounted for 19.04%, which is lower than the empirical standard point of 40%. Therefore, there was no serious common method variance in the current study.

### 3.2 Correlation analyses

**Figure 3:**
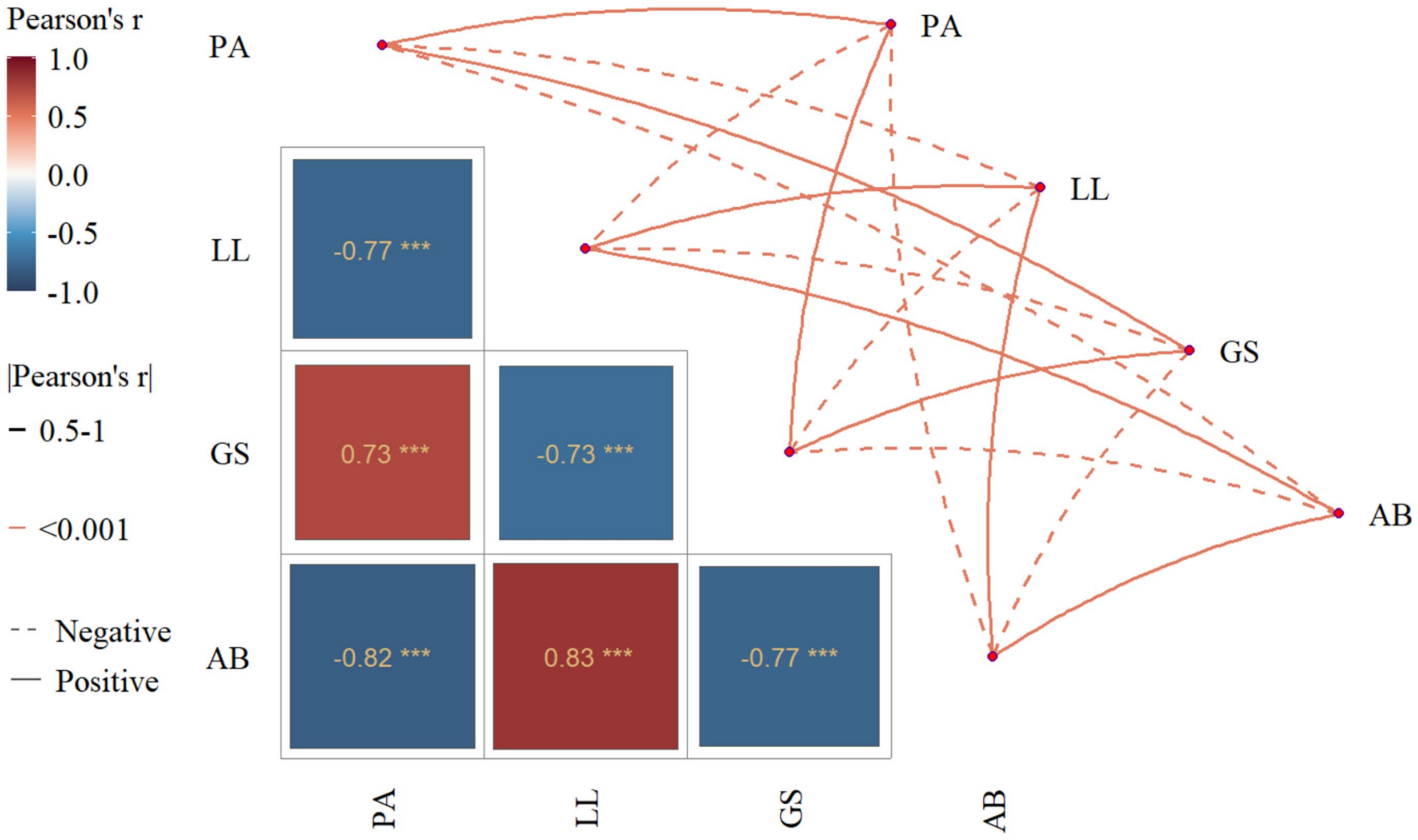
Mantel test of research variables.

Table 2 and Figure 3 present the bivariate correlations among physical activity, academic burnout, loneliness, and general self-efficacy. Physical activity was negatively correlated with loneliness (*r* = −0.769, *p* < 0.01) and demonstrated a strong negative association with academic burnout (*r* = −0.817, *p* < 0.01). A positive correlation was observed between physical activity and general self-efficacy (*r* = 0.733, *p* < 0.01). In contrast, both loneliness (*r* = −0.726, *p* < 0.01) and academic burnout (*r* = −0.775, *p* < 0.01) were negatively correlated with general self-efficacy. Additionally, loneliness was positively correlated with academic burnout. Supporting these findings, regression analysis in Table 3 identified physical activity as a significant negative predictor of academic burnout (*β* = −0.231, *p* < 0.001).

**Table 2:** Correlation coefficient matrix of research variables.

Variable	1. Physical activity	2. Loneliness	3. General self-efficacy	4. Academic burnout
1. Physical activity	1			
2. Loneliness	−0.769**	1		
3. General self-efficacy	0.733**	−0.726**	1	
4. Academic burnout	−0.817**	0.826**	−0.775**	1

^**^Indicates significance level *p* < 0.01.

**Table 3:** Regression analysis of the relationship between physical activity, loneliness, general self-efficacy and academic burnout.

Result variable	Predictive variables	*R* ^2^	*F*	Beta	*t*
Loneliness	Gender	0.593	92.864	0.775	0.989
	Age			0.058	0.266
	Physical activity			−0.489	−16.620
General self-efficacy	Gender	0.602	71.918	0.122	0.199
	Age			0.018	0.110
	Loneliness			−0.314	−5.554
	Physical activity			0.214	5.951
Academic burnout	Gender	0.788	140.077	−0.576	−0.937
	Age			−0.024	−0.138
	General self-efficacy			−0.334	−4.599
	Loneliness			0.419	6.872
	Physical activity			−0.231	−5.894

### 3.3 Bootstrap analysis of mediating effect significance test

#### 3.3.1 Mediating role of loneliness between the physical activity and academic burnout

Bootstrap analysis revealed a significant direct effect of physical activity on academic burnout (Effect = −0.302, 95%CI [−0.377, −0.228]). Furthermore, loneliness demonstrated a significant indirect mediating effect in the relationship between physical activity and academic burnout (indirect effect = −0.256, 95%CI [−0.369, −0.141]). Notably, the confidence intervals for both effects did not contain zero, and the effect sizes fall within an observable range, providing robust support for the proposed mediating model. These results indicate that loneliness functions as a partial mediator in the relationship. The proportion of this mediation relative to the total effect was 45.88%, suggesting that nearly half of the impact of physical activity on reducing academic burnout is explained through the mechanism of alleviating loneliness. The results are shown in Table 4.

**Table 4:** Loneliness bootstrap mediation effect tests.

Path	Effect size	BootSE	*t*	*p*	Bias-corrected 95%CI		Effect ratio
					Lower	Upper	
Total effect	−0.558	0.028	−19.667	<0.001	−0.615	−0.502	–
Direct effect	−0.302	0.038	−8.097	<0.001	−0.377	−0.228	54.12
Indirect effect	−0.256	0.056	–	<0.001	−0.369	−0.141	45.88

#### 3.3.2 Mediating role of general self-efficacy between the physical activity and academic burnout

The results support a model of mediation through general self-efficacy. This conclusion is grounded in the significant bootstrap estimates: an indirect effect of −0.191 (95%CI [−0.296, −0.109]) and a direct effect of −0.367 (95% CI[−0.441, −0.293]). The magnitude of these effects, which are substantial and whose confidence intervals exclude zero, confirms the mediating pathway’s empirical relevance. Quantitatively, this mediation accounts for 34.23% of the total effect, indicating that the enhancement of self-efficacy is a substantive mechanism underlying the relationship. For detailed results, see Table 5.

**Table 5:** General self-efficacy bootstrap mediation effect tests.

Path	Effect size	BootSE	*t*	*p*	Bias-corrected 95%CI		Effect ratio
					Lower	Upper	
Total effect	−0.558	0.028	−19.667	<0.001	−0.615	−0.502	–
Direct effect	−0.367	0.037	−9.825	<0.001	−0.441	−0.293	65.77
Indirect effect	−0.191	0.049	–	<0.001	−0.296	−0.109	34.23

#### 3.3.3 Mediating role of loneliness and general self-efficacy between the physical activity and academic burnout

**Figure 4:**
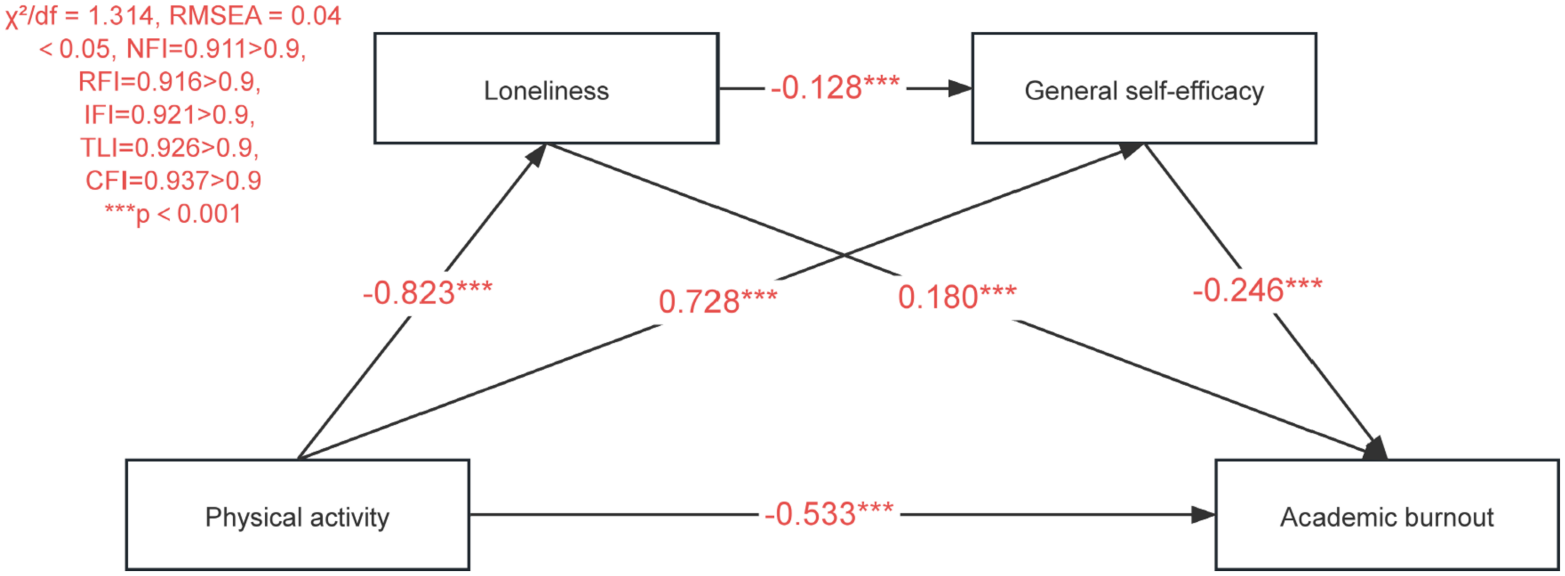
A chain-mediated model.

As shown in Tables 3, 6. We tested a chain mediation model examining whether loneliness and general self-efficacy sequentially mediate the relationship between physical activity and academic burnout. The model demonstrated excellent fit to the data (χ^2^/df = 1.314, RMSEA = 0.04, NFI = 0.911 > 0.9, RFI = 0.916 > 0.9, IFI = 0.921 > 0.9, TLI = 0.926 > 0.9, CFI = 0.937 > 0.9), as shown in Figure 4. All hypothesized regression paths were statistically significant (*p* < 0.05). Specifically, physical activity showed substantial negative associations with both loneliness and academic burnout, while loneliness negatively predicted general self-efficacy (*β* = −0.314), which in turn strongly predicted academic burnout (*β* = −0.334). Bootstrap analysis (*N* = 5,000) confirmed three significant mediating pathways. The indirect effect through loneliness alone was −0.205 (95%CI [−0.319, −0.097]), representing a moderate effect size according to psychological research standards. The pathway through general self-efficacy alone showed a smaller but meaningful effect of −0.071 (95%CI [−0.151, −0.025]). Most importantly, the sequential pathway through both mediators yielded an effect of −0.051 (95%CI [−0.093, −0.018]), indicating that the chained mediation, while modest in magnitude, represents a psychologically meaningful mechanism. The total indirect effect of −0.328 accounts for approximately 58.8% of the total effect (−0.558), indicating that over half of the association between physical activity and reduced academic burnout is mediated through the proposed chain of loneliness and self-efficacy. When considering both direct and indirect effects, the overall association corresponds to a large effect size (standardized coefficient = −0.558), highlighting the substantive importance of physical activity in mitigating academic burnout among left-behind children. These findings not only achieve statistical significance but also represent effects of meaningful practical magnitude for intervention design.

**Table 6:** An analysis of the chain-mediated effects of loneliness and general self-efficacy on the relationship between the physical activity and academic burnout.

Path	Effect size	BootSE	*t*	*p*	Bias-corrected 95%CI		Effect ratio
					Lower	Upper	
Total effect	−0.558	0.028	−19.667	<0.001	−0.615	−0.502	–
Direct effect	−0.231	0.039	−5.973	<0.001	−0.308	−0.154	41.40
Indirect effect	−0.328	0.062	–	<0.001	−0.455	−0.216	58.78
PA-LL-AB	−0.205	0.055	–	<0.001	−0.319	−0.097	36.74
PA-GS-AB	−0.071	0.033	–	<0.001	−0.151	−0.025	12.72
PA-LL-GS-AB	−0.051	0.019	–	<0.001	−0.093	−0.018	9.13

## 4 Discussion

### 4.1 Main findings

#### 4.1.1 Direct impact of physical activity on academic burnout

Employing a sequential-mediation framework, the study disentangled how physical activity attenuates academic burnout among China’s rural left-behind children. Findings reveal a clear, negative direct pathway from movement engagement to burnout, confirming Hypothesis 1 and resonating with an established corpus of cross-cultural research that positions physical activity as a salutary counter force to scholastic exhaustion (Feng et al., 2024; Zhu et al., 2023). This study verified that physical activity directly impacted the academic burnout among Rural Left-Behind Children in China.

#### 4.1.2 Mediating roles of loneliness and general self-efficacy

Supporting Hypothesis 2, the study positions loneliness as the operative mediator linking physical activity to academic burnout. Children whose movement is curtailed lack the embodied capital that active play confers, leaving them reliant on external validation and fostering a hyper-vigilant social schema in which interactions feel evaluative and unpredictable; this, in turn, crystallizes into chronic isolation. Echoing the Cognitive Discrepancy Model, loneliness is not simply a paucity of contact but a perceived deficit between desired and actual relational inputs—an interpretive gap widened by restricted physical activity (Rodriguez et al., 2025). Loneliness amplifies social-threat sensitivity (Leighton and Rudenstine, 2025), framing academic environments as adversarial and depleting motivational resources—thus serving as an affective mechanism linking physical inactivity to academic burnout. Physical activity indirectly mitigated academic burnout through reduced loneliness (*β* = −.256), it is mainly to make up for the lack of parent-child interaction to some extent by providing structured social interaction. This restoration of social connectedness disrupts the detrimental pathway from relational deficit to academic disengagement, reducing emotional exhaustion and renewing motivation. These results advocate for physical activity as a preventive strategy. Even moderate enhancements in social connectedness—facilitated through low-intensity school programs—may build psychological resilience and attenuate severe burnout (Plesko et al., 2021), informing scalable and practical mental health interventions in educational contexts.Hypothesis 3 is corroborated: general self-efficacy mediates the loneliness–burnout nexus. Echoing prior work, self-efficacy functions as an internal compass for negotiating academic demands; when this compass is weak, loneliness intensifies and burnout follows (Chen et al., 2025). Low self-efficacy undermines perceived competence, destabilizes self-concept, and impairs self-regulation in the face of academic demands—processes that magnify loneliness and, in turn, burnout. This pattern accords with social cognitive theory, which positions self-efficacy as a core mechanism sustaining motivation and resilience (Setyaningrum Weny et al., 2024). Elevated self-efficacy engenders an internal locus of control over academic outcomes and elicits affirmative feedback loops, collectively safeguarding well-being and dampening burnout. Consequently, self-efficacy emerged as a pivotal cognitive mechanism mediating the influence of loneliness on academic burnout. The model revealed a significant indirect pathway from physical activity to reduced academic burnout through enhanced self-efficacy (*β* = −0.191). A simulated one-standard-deviation increase in self-efficacy potentially attainable through integrated home-school interventions—corresponded to an estimated 1.9-point decrease in loneliness scores (Xuto et al., 2024). Although marginally below the conventional clinical benchmark of a 2-point reduction, this finding underscores self-efficacy as a modifiable and pragmatically meaningful target for intervention.

#### 4.1.3 Chain mediating roles of loneliness and general self-efficacy

Beyond its direct salutary influence, physical activity exerts an indirect, sequential effect on academic burnout through the linked mediators of loneliness and general self-efficacy, thus confirming Hypothesis 4 and corroborating prior evidence that loneliness forecasts heightened burnout risk (Khurshid et al., 2025). Empirical evidence indicates that loneliness prospectively forecasts elevated academic burnout; perceived social isolation erodes motivational resources and curbs scholastic engagement. General self-efficacy, in turn, operates as a pivotal determinant of both loneliness and burnout: children endowed with robust self-efficacy exhibit attenuated loneliness and, consequently, diminished academic exhaustion. Children with higher general self-efficacy are less likely to experience loneliness and academic burnout (Malakcioglu, 2024). Heightened self-efficacy equips pupils with the perceived competence to navigate academic demands, fostering adaptive social exchanges and superior scholastic outcomes; conversely, attenuated efficacy intensifies loneliness and, by extension, burnout. Our sequential-mediation analyses reveal that sustained physical activity first fortifies general self-efficacy among rural left-behind children, which in turn attenuates loneliness, culminating in diminished burnout. This affective-cognitive chain functions as a partial, yet robust, pathway linking movement behaviors to reduced academic exhaustion. These insights delineate the mechanistic circuitry through which exercise safeguards academic well-being and inform targeted interventions that simultaneously cultivate self-efficacy and curtail loneliness, thereby enhancing both scholastic performance and psychological health in this vulnerable population.

A key limitation of this study is its cross-sectional design. Since all variables—physical activity, loneliness, general self-efficacy, and academic burnout—were measured at the same time, we cannot draw causal conclusions about their relationships. For example, the negative correlation between physical activity and burnout could mean either that exercise reduces burnout, or that less burnt-out students are more likely to be active. Similarly, the temporal order of self-efficacy as a mediator remains unclear. Reverse causality is also possible, as severe academic burnout may lead to social withdrawal (increased loneliness), lower activity motivation, and reduced self-efficacy. Although the statistical results support the hypothesized mediation model, other explanations—including bidirectional effects—cannot be excluded. Therefore, the proposed model should be viewed as a theoretically and empirically grounded set of associations, not a confirmed causal pathway. Still, this study offers a useful basis for understanding these complex interactions. Importantly, the main pathways identified are consistent with several longitudinal studies, which lends indirect support to the model and provides a solid starting point for future research using longitudinal or experimental designs to establish causality.

### 4.2 Implications of this research

This study clarifies both the direct pathway linking physical activity to academic burnout among China’s rural left-behind children and the sequential mechanisms through which loneliness and general self-efficacy transmit this effect. By integrating Conservation of Resources and Social Cognitive perspectives, the findings advance theory on how movement behavior intersects with socio-cognitive and affective processes to shape school-related well-being. Empirically, regular physical activity emerges as a salient protective factor that dampens burnout risk. Beyond this direct benefit, loneliness and self-efficacy operate as serial mediators: physical activity first attenuates loneliness, thereby fortifying general self-efficacy, which in turn reduces burnout. These insights suggest that multi-component interventions—simultaneously curbing loneliness and cultivating self-efficacy—can substantially enhance left-behind children’s stress-adaptation capacities, ultimately improving both psychological functioning and academic trajectories.

### 4.3 Limitations

There are four limitations in this study. First, although theoretically grounded and tested using mediation analysis, the cross-sectional nature of this study precludes definitive causal conclusions. The proposed pathways (physical activity → loneliness → general self-efficacy → academic burnout) reflect a theoretically informed statistical model rather than demonstrated causality. Future longitudinal or experimental studies are essential to confirm the temporal precedence and causal direction of these relationships. Second, although the sample demonstrates adequate statistical power and provides valuable insights into a specific regional context, its restriction to a single county in Hunan Province limits the generalizability of the results. Future research incorporating samples from diverse socioeconomic regions across China would help verify the broader applicability of the findings. Third, self-reported data may inflate effect sizes despite mitigation measures; objective or multi-source measurements are recommended for future research. Finally, the omission of socioeconomic status (SES) as a moderator constrains theoretical insight; testing moderated mediation models with composite SES indicators would help identify boundary conditions and enhance mechanistic understanding.

## 5 Conclusion

This cross-sectional study finds that physical activity not only directly predicts academic burnout among rural left-behind children but also indirectly influences. It through the independent mediating effects of loneliness and general self-efficacy, as well as the chain-mediating effect of loneliness on general self-efficacy. This study reveals the mechanism through which physical activity impacts academic burnout among rural left-behind children from both emotional (loneliness) and psychological (general self-efficacy) perspectives. Accordingly, promoting physical activity, reducing feelings of loneliness, and enhancing general self-efficacy can potentially alleviate academic burnout among rural left-behind children. These findings suggest that interventions aimed at increasing physical activity may not only directly reduce academic burnout but also indirectly improve children’s psychological resilience through enhanced self-efficacy and reduced loneliness.

## Data Availability

The original contributions presented in the study are included in the article/supplementary material, further inquiries can be directed to the corresponding authors.
